# Emergency Floor Plan Digitization Using Machine Learning

**DOI:** 10.3390/s23198344

**Published:** 2023-10-09

**Authors:** Mohab Hassaan, Philip Alexander Ott, Ann-Kristin Dugstad, Miguel A. Vega Torres, André Borrmann

**Affiliations:** Chair of Computational Modeling and Simulation, Technical University of Munich, 80333 Munich, Germanymiguel.vega@tum.de (M.A.V.T.);

**Keywords:** emergency floor plans, object detection, machine learning, faster R-CNN, synthetic data

## Abstract

An increasing number of special-use and high-rise buildings have presented challenges for efficient evacuations, particularly in fire emergencies. At the same time, however, the use of autonomous vehicles within indoor environments has received only limited attention for emergency scenarios. To address these issues, we developed a method that classifies emergency symbols and determines their location on emergency floor plans. The method incorporates color filtering, clustering and object detection techniques to extract walls, which were used in combination to generate clean, digitized plans. By integrating the geometric and semantic data digitized with our method, existing building information modeling (BIM) based evacuation tools can be enhanced, improving their capabilities for path planning and decision making. We collected a dataset of 403 German emergency floor plans and created a synthetic dataset comprising 5000 plans. Both datasets were used to train two distinct faster region-based convolutional neural networks (Faster R-CNNs). The models were evaluated and compared using 83 floor plan images. The results show that the synthetic model outperformed the standard model for rare symbols, correctly identifying symbol classes that were not detected by the standard model. The presented framework offers a valuable tool for digitizing emergency floor plans and enhancing digital evacuation applications.

## 1. Introduction

Increasing urbanization and urban densification, as well as the resulting trend of building upwards, has led to a substantial increase in special-use and high-rise buildings [1]. These structures present greater complexity, posing challenges for quick and well-organized evacuations, particularly in fire emergencies. At the same time, advancements in technology enable the use of autonomous vehicles, such as unmanned aerial vehicles (UAVs) and unmanned ground vehicles (UGVs) within indoor environments. As stated in [2], they have been given too little attention for emergency scenarios so far.

These evacuation issues have been recognized and the associated challenges have prompted research efforts to identify optimal evacuation strategies [1,3,4]. As [4] demonstrated in their study, a comprehensive understanding of the building’s structure, combined with situational awareness, can significantly enhance the efficiency and time effectiveness of evacuations. To this end, path-planning tools are currently being developed to provide support in evacuation scenarios and reduce the emergency response time, in turn increasing the efficiency of first responders. The strategies developed so far use geometric data of buildings combined with real-time information on hazards [1,2,5,6]. However, none of these models utilize emergency floor plans, which provide publicly accessible information on a building’s structure, as well as essential firefighting procedures. Escape routes, relevant firefighting equipment and first-aid resources are visualized in these plans, supporting evacuees, emergency responders, and firefighters alike.

To close this gap, we have developed and present a method that classifies emergency symbols and determines their location on emergency floor plans. For the category-specific object detection (COD) task, a Faster R-CNN was employed. The extraction of walls from the floor plans is facilitated by color filtering and clustering techniques, resulting in clean plan images. While our Faster R-CNN adheres to the model architecture originally proposed by [7], this study is characterized by the creation of a comprehensive dataset, the development of a workflow for generating synthetic emergency floor plans, and the implementation of a pipeline for generating clean plan images. By incorporating geometric and semantic information from emergency floor plans, existing BIM based evacuation applications, such as path-planning tools, can be enhanced, thereby further improving their capabilities. As highlighted by [8] in their review, there is still a lack of semantic data through object detection in BIM. The method can be applied in emergency situations, where the scan of an emergency floor plan, followed by automatic evaluation, enables UAVs and UGVs to find escape routes. The benefits of the presented method extend beyond emergencies by providing maintenance personnel and fire protection authorities comprehensive overviews of available resources and evacuation pathways, enhancing their planning and decision-making process.

The extraction of architectural features has been extensively researched [9,10,11,12]. Hence, for training a model detecting walls and other architectural structures in 2D plans, large-scale open-source data sets are already available [13]. In contrast, the detection of emergency symbols on plans supported by object detection has not yet been investigated, and no open-source data set with emergency floor plans is available. A comprehensive database was thus set up for training, validating, and testing the neural networks. A total of 403 emergency floor plans, mainly photographs, were collected in public buildings located in Germany. For the COD task, the emergency symbols were labeled using bounding box rectangles. Considering the limited availability of collected plans, an additional dataset was synthetically created. By manipulating 200 of the gathered plans, a new dataset comprising around 5000 plans was generated. Subsequently, this dataset was employed to train a distinct Faster R-CNN model, the so-called synthetic model. Notably, our findings demonstrate that the synthetic model outperforms the standard model trained on the original dataset in the detection of certain emergency symbols. Furthermore, it is worth highlighting the comparatively lower effort required to generate synthetic data, relative to the labor-intensive process of collecting and labeling additional emergency floor plans.

In the following section, prior research concerning the digitization of 2D floor plans is presented. In Section 3, we introduce our methodology, as well as the method’s framework providing a comprehensive insight into the employed neural networks (NNs). In Section 4, we present the generated dataset and detail the creation of the synthetic data. Subsequently, the results of our two approaches, namely the model trained with the collected emergency floor plans, as well as the model trained with the synthetic dataset, are discussed in Section 4.4. The paper concludes with a summary of the findings. Moreover, we give an outlook on potential application domains, along with suggestions for enhancements and future prospects.

## 2. Related Work

Digital tools such as computer-aided design (CAD) have emerged relatively recently, and many existing building plans, including emergency floor plans, are available only in a non-digitized format. Consequently, several studies have focused on the digitization of floor plans to enable the extraction of information for further processing and analysis. As early as 2009, ref. [14] conducted a survey on the generation of 3D building models from 2D architectural drawings. Ref. [10] introduced a research work addressing the same objective. Their initial step involves preprocessing the 2D architectural plan to remove noise and unnecessary information by binarizing the image. It is then separated into two components: a text image containing textual information and a geometry image containing geometrical elements. The text image is analyzed using optical character recognition (OCR) techniques; the geometry image is processed to find geometry primitives such as segments and arcs using the Hough transform method.

In 2022, ref. [15] introduced a method improving instance segmentation of architectural floor plans using machine learning. They also provide a good review of feature extraction of 2D floor plans. State-of-the-art methods include the use of NNs for segmentation, such as walls, and object detection. Many researchers have addressed the development, performance, and use cases of segmentation models [16,17,18]. Ref. [19] has particularly studied the UNet model and shown that it performs well in image processing. Object detection, and COD in particular, have become an integral part of image processing. There are several extensive reviews on these models’ roles, use cases, limitations and future perspectives [20,21].

In their study, the authors of ref. [9] employ a combination of three methods, namely wall segmentation, object detection, and OCR, to extract both geometric and semantic information from 2D floor plans. For wall segmentation, fully convolutional networks (FCNs) are utilized. Additionally, a patch-based approach for wall detection is implemented. Regarding object detection, the Faster R-CNN framework is employed. A subset of the R-FP dataset is annotated with six different object classes. The model is trained on only 144 images for 150 epochs. Similarly, ref. [22] employed convolutional neural networks (CNN) for floor plan digitization. Their approach involved the utilization of a UNet model for wall segmentation and a Faster R-CNN for detecting architectural elements such as doors and windows. Their dataset consists of 700 floor plans. The authors employed an augmentation process to increase their dataset, resulting in a total of 2000 floor plans. However, despite the augmentation, the images in the dataset still exhibited significant similarity. In a related study, the authors of ref. [12] conducted floor plan digitization using DeepLab3+ for wall segmentation and YOLOv4 for object detection, along with OCR. Their dataset was substantially larger, consisting of 7000 floor plans, with 5600 used for training purposes.

The presented approaches are closely related to the pipeline presented in this paper. However, their approaches were never conducted on emergency floor plans. The detection task is made for standard symbols in architectural floor plans like walls, doors and windows. The total number of object classes in these projects is small. In this study, a holistic approach was taken to capture each relevant floor plan symbol for evacuation scenarios, adding up to a total of 56 object classes. The inclusion of this large number of symbol classes, some of which exhibit significant visual similarity, combined with the challenging data situation necessitated the development of a synthetic data set.

An investigation into the performance of the Cascade Mask R-CNN network for object detection in floor plan images was conducted by [23]. They proposed the incorporation of deformable convolution and introduced a synthetic floor plan dataset (referred to as SFPI) for the purpose of training and evaluation. For the generation of synthetic data, the authors created a dataset consisting of 10,000 images. This dataset encompassed ten distinct floor plan layouts, with each layout incorporating a straightforward insertion of furniture objects, divided into 16 distinct furniture classes. In comparison, the original dataset comprised 700 samples for training and 150 samples for testing. On the other hand, the synthetic dataset consisted of 7000 samples for training and 1500 samples each for validation and testing, facilitating robust evaluation of the proposed method’s performance. However, only 16 object classes of widely differing symbols were used. Since the extraction of architectural elements, namely walls and staircases, was also goal of this study, the repetitive use of only 10 floor plans left doubts about the effective training of neural networks using this method.

In a recent study, ref. [24] address the challenge of acquiring large and accurately labeled datasets for training neural networks. The framework employs a data augmentation technique called random cropping to create a complete and automatically labeled dataset that can be directly used as input for deep learning models. The study also investigates the significance of context in image recognition, particularly the influence of the relative resolution between symbols and the background image. The authors demonstrate their algorithm’s effectiveness by focusing on the detection of a valve symbol as a proof-of-concept. Their concept has already been adapted by [25] and successfully applied for the digitization of bridge construction plans.

In contrast to previous approaches, our project addresses the specific challenge of digitizing emergency floor plans, which are often only available in 2D format. While previous studies have focused on the extraction of information from architectural floor plans, our approach aims to capture a larger number of emergency relevant floor plan symbols, incorporating a comprehensive set of 56 object classes. For this purpose, we incorporate a synthetic data set inspired by the work of [24], encompassing ca. 5000 images. This large and diverse dataset allows for more effective training of our neural network.

## 3. Methodology

**Figure 1:**
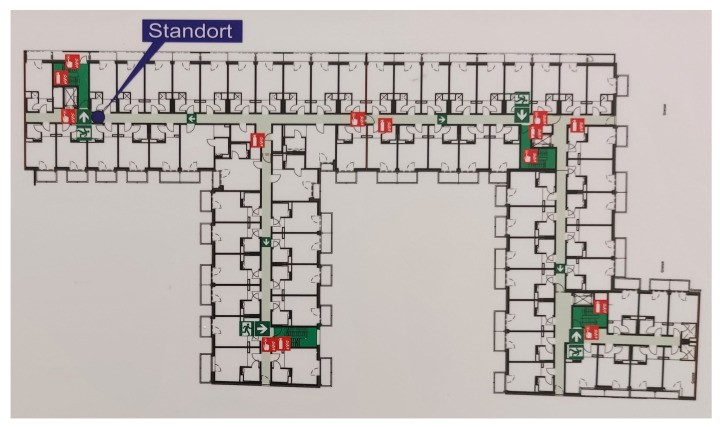
Sample emergency floor plan image. Note: All non-relevant sections of the floor plan, such as the legend, have been manually cropped.

The use of NNs in the field of computer vision is well recognized; numerous studies have been previously published, demonstrating their robustness and reliability. The aim of this study is to investigate the feasibility of using NNs to digitize emergency floor plans. A sample image of an emergency floor plan is presented in Figure 1.

To successfully digitize emergency floor plans, all emergency symbols, staircases and building walls must be correctly extracted, with the latter’s only use being to recreate a digital version of the original plan, either as a vector file or a high-resolution image. In this study, the output is a high-resolution PNG image.

**Figure 2:**
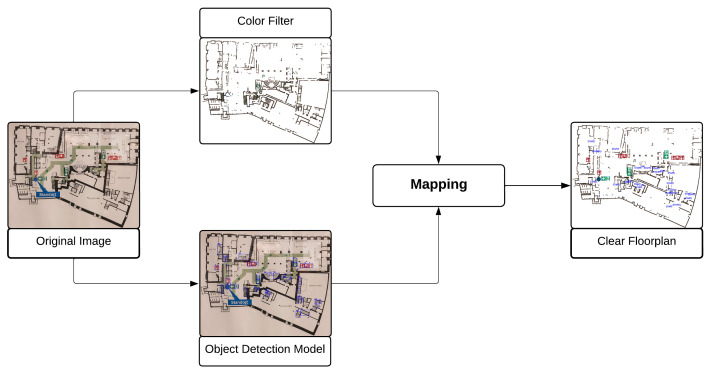
General pipeline used within this study. The original image is fed into the category-specific object detection (COD) and CF. The outputs of both models are then mapped together to create a clear floor plan, i.e., the clean plan discussed in Section 3.

The general pipeline developed for this study is depicted in Figure 2. Here, two types of models were implemented to extract the emergency symbols and walls contained within images of emergency floor plans. In this study, these models are referred to as the category-specific object detection (COD) model and the color filter (CF) model. The models are discussed in Section 3.1 and Section 3.2, respectively.

For a given emergency floor plan, the location and type of all emergency symbols are extracted by the COD model, while the walls are extracted by the CF model. The output of the CF model is used to recreate a *clean plan*. This clean plan is then overlaid with images of symbols in the locations detected by the COD model.

**Figure 3:**
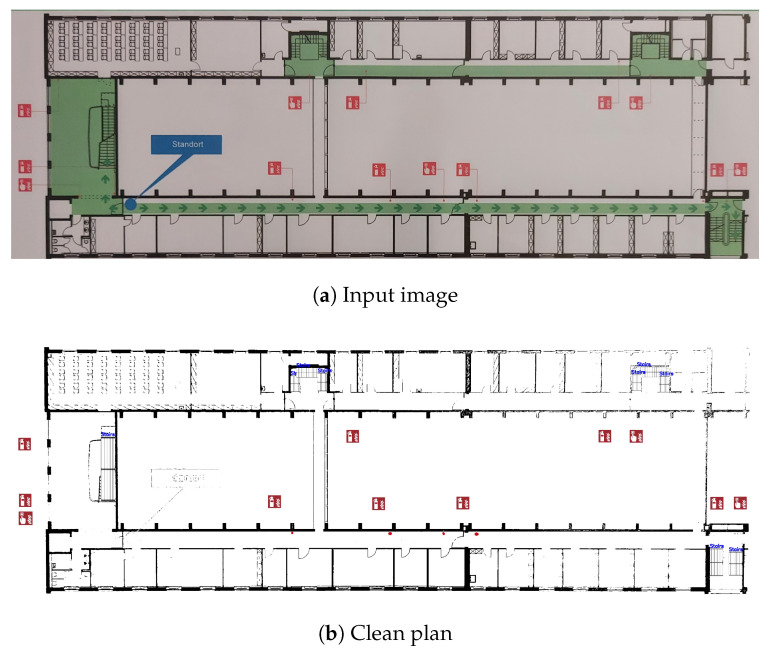
The input and output images of the pipeline presented in this study. (**a**) The input of the pipeline: a typical emergency floor plan. (**b**) The output of the pipeline: the original image’s corresponding clean plan. Note: The input image has been manually cropped to remove all non-relevant information.

In this paper, the term “clean plan” refers to a digitized, high resolution version of an emergency floor plan. In such plans, all information, with the exception of the emergency symbols, as well as walls and staircases, is omitted. A comparison between a typical emergency floor plan and a clean plan is presented in Figure 3.

### 3.1. Object Detection Model

In this study, the COD model is used to identify the location of all emergency symbols within an emergency floor plan. The expected output of this model is a set of bounding box and class pairs. In the context of this study, a class refers to the name of an emergency symbol. Further discussion on symbol classes is provided in Section 4.1.1.

The output bounding boxes are rectangular and are defined by the coordinates of two vertices, representing the lower-left and upper-right corners of the rectangle. The coordinates can alternatively be interpreted as the lower and upper bounds of the rectangle in each direction. This bounding box’s definition is shown in Equation (1), where the subscripts ${}_{min}$ and ${}_{max}$ represent the lower and upper bounds, respectively.

(1) $$bounding\;box=[x_{min},\;y_{min},\;x_{max},\;y_{max}]$$

The neural network architecture employed in this study is the Faster R-CNN, a combination of a Regional Proposal Network (RPN) and a ResNet-50-FPN. More information on Faster R-CNNs can be found in [7]. The loss functions utilized by both networks are summarized in Table 1.

Emergency floor plans tend to become crowded with symbols, especially in larger buildings where the information is crucial for efficient evacuation processes. Accurate detection and localization of small symbols holds significant importance in such cases. In this regard, the Faster R-CNN outperforms alternative models, such as you-only-look-once (YOLO) and single-stage detection (SSD), as was shown in [26,27,28], deeming it an appropriate choice for the COD model.

A Stochastic Gradient Descent (with momentum) optimizer was used to train this model. In addition to an $L_{2}$ penalty of $5\times 10^{-4}$, a learning rate (α) and momentum factor (β) equating to $1\times 10^{-3}$ and 90% were used as the hyperparameters. The formulation of this optimizer can be found in [29].

The input to this model is an RGB image with the shape [width,height,channels]=[416,416,3], where *channels* denotes to the number of channels (3: red, green and blue). It should be noted that the input dimensions were chosen as such due to the available computational resources, and can be thought of as an arbitrary choice.

### 3.2. Color Filter Model

**Figure 4:**
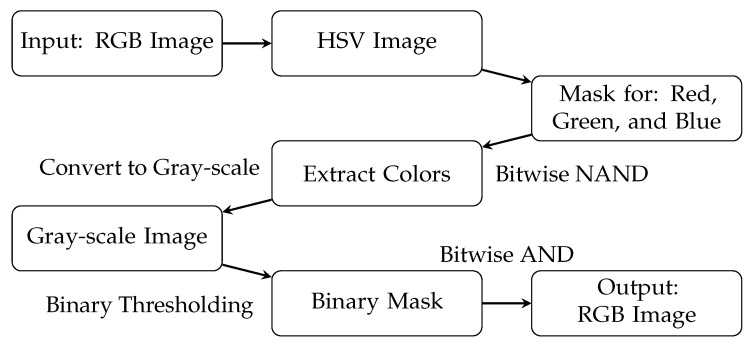
Description of the CF model. The input to this model is an RGB image. The output is an RGB image of all solid, black lines in the input image.

In this study, the CF model is used to extract all walls from the emergency floor plan. Here, a non-trainable color filter was employed for this task. The step-by-step procedure is presented in Figure 4, and the values used to create the masks in HSV space are presented in Table 2. The masks were subsequently merged together using the binary OR operation.

The output of this model is a PNG image of the same shape as the input image—an RGB image in this case—containing all solid, black lines. All non-black pixels are replaced by white pixels, i.e., pixels with the following values: [red,green,blue]=[255,255,255].

## 4. Experiments and Results

### 4.1. Datasets

The detection of emergency symbols on floor plans, facilitated by object detection techniques, has not been investigated before. There are therefore no open-source emergency floor plan datasets for object detection models. In order to address this gap and provide valuable resources for future investigations, two distinct datasets were created for the purpose of this study.

The first dataset consists of 403 German emergency floor plans, mainly photographs. The focus on German plans was due to the significant heterogeneity in styles and formats of emergency floor plans worldwide. This dataset was used for training and validation of the standard model, as well as for testing both the standard model and the synthetic data model. The second dataset was utilized for training the synthetic data model. A total of approximately 200 emergency floor plans were employed to generate the synthetic dataset, yielding approximately 5000 training images. The process with which the synthetic dataset was created is presented in Section 4.2.

#### 4.1.1. Relevant Symbols

A comprehensive list of all emergency symbols, found in the dataset mentioned in the preceding paragraph, was compiled. This list can be found in Appendix A. A total of 45 relevant symbols with different functions were identified. Twelve of them exist in two different graphical styles. This duality can be attributed to the presence of two standards governing emergency symbols and floor plans in Germany: the former German standard DIN 4844-2 and the currently valid DIN EN ISO 7010, which has been in effect throughout Europe since January 2013 [30]. Despite the ten-year duration of the DIN EN ISO 7010 standard, considerable heterogeneity persists among European plans, and a significant proportion of old-style plans are still prevalent in Germany. A notable example of this heterogeneity is the circular symbol denoting to the current location of an individual, for which no universally accepted color convention has been established.

#### 4.1.2. Labeling

To train the neural networks and analyze their performance, it is essential to label the entire data set for providing ground truth. This process ensures that the data are properly categorized and serves as a benchmark for evaluating the performance of the networks. The open-source tool LabelImg was used to label the plans for the COD. It allows easy management of many classes and annotations using bounding boxes, which is fully sufficient for emergency symbols, as the majority of symbols in the data set have a square shape. Other symbols like circles and equilateral triangles can easily be enclosed in a rectangle and their position can be determined exactly. The output is provided as XML files in PASCAL VOC format, containing all relevant information and allows simple further processing.

In an iterative procedure, the number of training and validation plans was successively increased. Approximately 20% of the plans underwent manual labeling, which was necessary to establish an initial training state of the model. The model was then used to pre-label plans that had not yet been annotated, thereby significantly expediting the labeling process. This approach proved to be particularly advantageous when the model had already achieved some level of proficiency through training. However, it should be noted that, in addition to incorporating the labels of undetected symbols, all pre-labels were carefully examined for incorrectly identified symbols and imprecise bounding boxes. During the initial stages, this verification process increased the effort needed; however, the overall effort of this semi-automatic labeling process was significantly lower, particularly during later iterations.

### 4.2. Preprocessing—COD Model

As mentioned in Section 3.1, the input shape for the COD model is [416,416,3]. Consequently, each image in the training dataset is resized to match the input shape before training. This change in shape, however, leads to a modified image with distorted symbols. Such an effect is amplified for plans that deviate from a square aspect ratio, i.e., an aspect ratio of unity. In this study, the aspect ratio of an image here is defined as the ratio of its dimensions: $\frac{width}{height}$.

**Figure 5:**
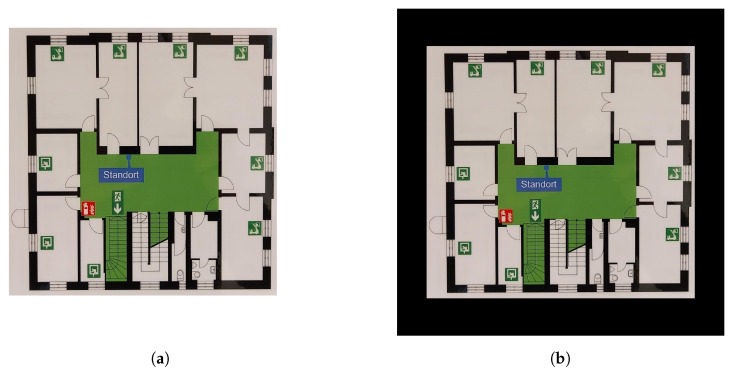
A comparison between the emergency floor plan image before applying zero-padding (**a**) and after applying it (**b**).

To overcome this issue while preserving the diversity in symbol sizes, zero-padding was applied to all training images. The padded images have a shape of [d,d,3], where *d* is the largest dimension found among all training images. Figure 5 presents a sample emergency floor plan before and after applying zero-padding.

In addition to zero-padding, various data augmentation operations were applied onto the training dataset. These operations are detailed in Table 3, where *p* is the probability of applying each transformation to an individual plan in the dataset.

Each transformation was applied sequentially, i.e., the probability of applying all transformations to a single plan is $\prod_{i=1}^{n}p_{i}$, where *n* is the number of transformations. It should be noted that the list of transformations, described in Table 3, is applied to the unaltered dataset at the start of each epoch, i.e., the dataset is reset to the pre-transformation state before reapplying all five operations.

#### Synthetic Data

**Figure 6:**
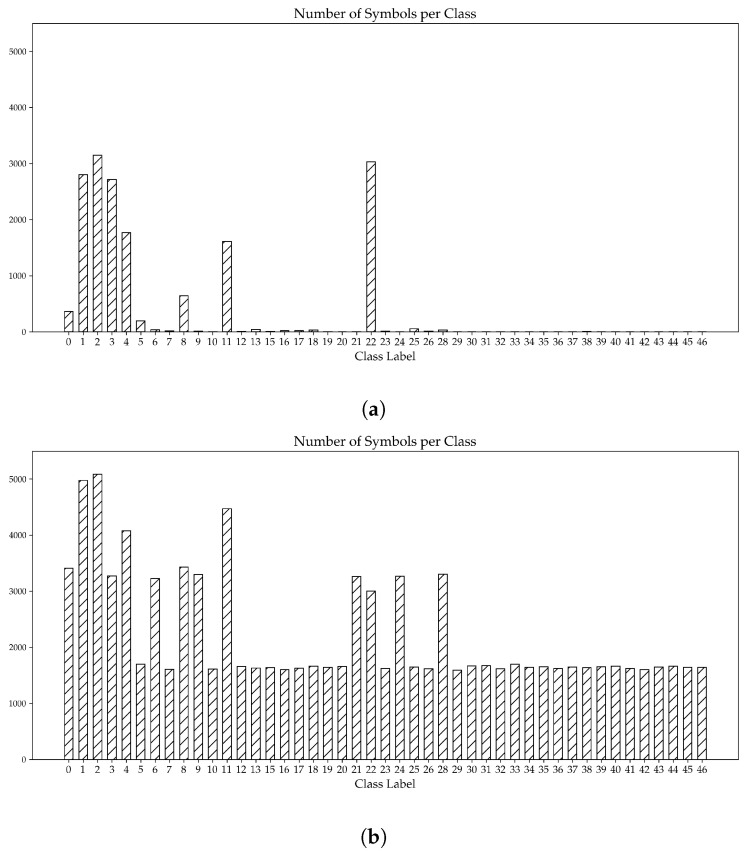
Distribution of symbols across both the original dataset (**a**) and the synthetic dataset (**b**). The x-axis represents all the symbol classes considered within this study, while the y-axis presents the number of symbols per symbol class. Note: All symbol classes can be found in Appendix A. Note: To avoid clutter, all versions of the same symbol have been merged into one symbol class.

An initial assessment of the training dataset (discussed in Section 4.1) highlights a notable disparity in the number of symbols for each symbol type, as can be seen in Figure 6a. This disparity arises from the fact that certain symbols are not commonly found in all buildings; rather, they are exclusive to specific types of buildings. For instance, escape hatch symbols (symbol number: 16_4844-2) only occur in two plans in the dataset. Furthermore, this disparity is more prominent for specialized symbols, such as the emergency shower (symbol number: 15), which are only found in rooms designated for the handling of caustics.

**Figure 7:**
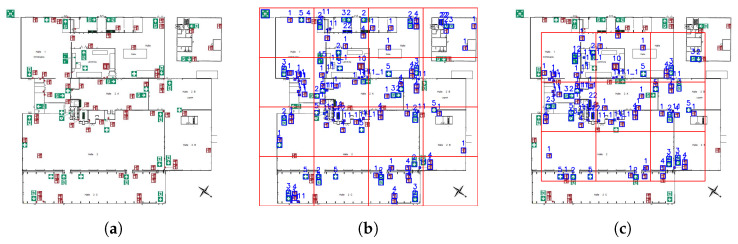
The synthetic dataset workflow. (**a**) The original emergency floor plan image. (**b**) The first set of cutouts: 16 images in total. (**c**) The second set of cutouts: nine images in total.

To minimize the imbalance depicted in Figure 6a, a synthetic dataset was created from the original dataset. The general idea, adapted from [24], is presented in Figure 7. Each emergency floor plan in the training dataset was initially cut into 25 equally sized sub-images, hereafter referred to as cutouts.

The cutouts were created in a two-step process. First, the original image—with a shape of [w,h,3], where *w* and *h* are the image’s width and height, respectively—was divided into 16 cutouts with a shape $=[\frac{w}{4},\;\frac{h}{4},\;3]$. This process was then repeated on the same image to create nine additional cutouts, albeit on a sub-image defined by the bounding box $\left[x_{min},\;y_{min},\;x_{max},\;y_{max}\right]=\left[\frac{w}{8},\;\frac{h}{8},\;\frac{7w}{8},\;\frac{7h}{8}\right]$, where *w* and *h* represent the image’s width and height, respectively. The original image’s corresponding labels were then mapped onto the new cutouts, resulting in 25 pairs of XML and JPG files, for each emergency floor plan image.

Synthetic symbols, i.e., high-resolution images of symbols, were then overlaid onto all cutouts at random locations. This process was performed in a recursive manner, where a location was assigned at random, and was only used if the symbol’s bounding box did not intersect with another symbol’s bounding box. This recursive algorithm was limited to 100 iterations, after which, if an intersection-free location was not found, the symbol was skipped.

To avoid overcrowding a cutout, the number of synthetic symbols per cutout was limited to 12 and was calculated using Equation (2), where $N_{synthetic}$ and $N_{original}$ are the number of synthetic and original symbols in a cutout, respectively.

(2) $$N_{synthetic}=\left\{\begin{array}{cc} 12-N_{original} & \mathrm{if}\;N_{original}<12\\ 0 & \mathrm{otherwise} \end{array}\right.$$

All synthetic symbols were initially resized and blurred, before being overlaid onto the cutouts. To preserve the original diversity in symbol sizes, the dimensions of all synthetic symbols were determined by taking the mean of the width and height of all the original symbols within each cutout. Furthermore, the symbols were blurred using a Gaussian blur, with a kernel size of [5,5]. The blur variance for each symbol was estimated by applying a Laplacian operation to each cutout.

Half the training dataset (200 emergency plans) was used to create the synthetic dataset, resulting in 5000 pairs of XML files and JPG images. A fifth of those JPG/XML pairs were used as the validation dataset, while the rest was used as the training dataset.

Figure 6b shows the distribution of symbols across all symbol types. It is evident that the imbalance, observed earlier in the distribution of symbols in the original dataset (Figure 6a), has been minimized. For the remainder of this paper, the COD model, which was trained using the synthetic dataset, will be referred to as the synthetic model, while the model trained using the original dataset will be referred to as the standard model.

### 4.3. Preprocessing—CF Model

Within this study, the objective of the CF model is to extract all walls from the emergency floor plan dataset. These walls are then overlaid with the predictions of the COD model to generate clean plans.

Generally, all walls in an emergency floor plan are solid, black lines of varying thicknesses. Therefore, a k-means clustering algorithm (k=4) was used as a pre-filter to remove all non-relevant data from the image: all non-black pixels in the image; the first three clusters. The output of this algorithm was then fed into the CF model.

### 4.4. Inference—COD Model

The trained object detection models, the standard model and the synthetic model were tested using 83 emergency floor plan images: those not used in during the training of the standard model. The images were initially padded in a similar manner to the one described in Section 4.2. Aside from resizing the padded, inference images to the expected input shape of the COD models, [width,height,channels]=[416,416,3], no preprocessing steps were performed on the images.

For both models, detections with a confidence score lower than 70% were excluded. Moreover, if the intersection-over-union value of two predicted bounding boxes was higher than 90%, the detection with the lower confidence score was filtered out. The complete inference pipeline, including both the preprocessing of the inference image and the postprocessing of all detections, took an average time of 0.075 s, corresponding to an average FPS of 13.328. The machine’s specifications are presented in Table 4.

**Figure 8:**
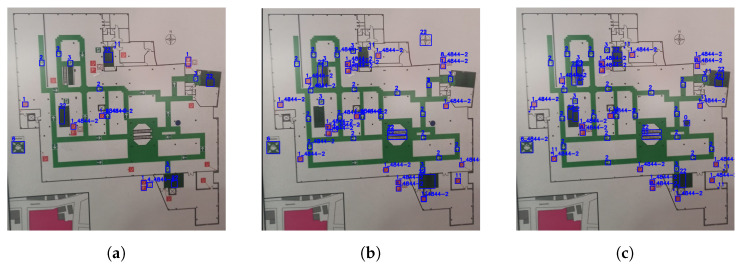
Visualization of the detections of both the standard model (**a**) and the synthetic model (**b**) on a sample emergency floor plan. Both detections are compared to the ground truth (**c**). The detections are presented as pairs of a blue, rectangular box bounding the location predicted by the model, and a class label (blue font). For interpretation of the class labels, refer to Appendix A.

The detections (also called predictions) of both models were compared to their corresponding benchmark labels, i.e., their manually labeled counterparts. Within the remainder of this section, these benchmark labels will be referred to as the ground truth. Figure 8 presents the detections of both the standard and synthetic models, on a sample emergency floor plan (Figure 8a,b, respectively), as well as the corresponding ground truth labels (Figure 8c).

Generally, the number of correct detections, generated by the synthetic model, surpasses the ones generated by the standard model. This is due to the substantially larger number of symbols per class present in the synthetic dataset.

Moreover, a detailed analysis of the direction arrow symbols (symbols 2 and $2^{*}$) reveals the standard model’s susceptibility to variations in background conditions. While the standard model is proficient at detecting these symbols, it struggles to detect them in areas where the background color is similar to the symbol’s color. This limitation can be seen in Figure 8a, where only 4 out of the 17 direction arrows located in front of a green background have been detected, in contrast to the synthetic model, where 16 direction arrows were detected (Figure 8b).

An exception to the synthetic model’s higher performance is the stairs symbol (symbol 22), where its relatively simple geometry has led to overfitting. Since the synthetic model is trained on cutouts with overlaid images of symbols, it exhibits difficulties in accurately detecting stairs with varying geometry and background color. The symbol images used to create the synthetic dataset can be found in Appendix A (refer to Section 4.2). This issue has additionally resulted in several false detections throughout the inference dataset. For instance, the model incorrectly identifies a navigation symbol as a staircase.

**Figure 9:**
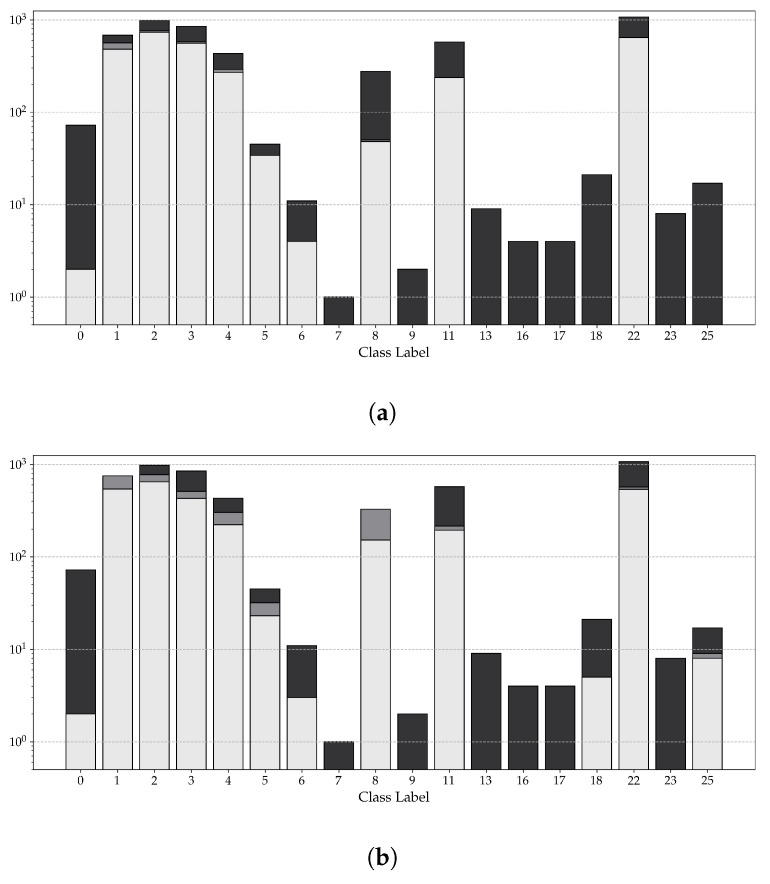
Visualization of the the number of undetected symbols (solid black bar), the number of incorrect detections (dark shaded gray bar) and the number of correct detections (light shaded gray), presented for both the standard model (**a**) and the synthetic model (**b**). Notes: Only classes that occur at least once throughout the inference dataset are presented. To avoid clutter, all versions of the same symbol have been merged into one. The y-axis in both subfigures is a logarithmic scale.

Figure 9 visualizes the number of correct and incorrect detections, as well as the number of undetected symbols, for both the synthetic and standard models. A clear distinction can be seen when examining both models’ performance for common and rare symbols, defined as symbols that occur more than 300 times and less than 50 times in the inference dataset, respectively.

For common symbols, the performance of both models is comparable, with the only difference being the synthetic model’s higher number of total detections relative to the standard model. However, a clear difference in performance can be seen for the rare symbols. For instance, ∼50% of all symbols of the classes 18 and 25 were detected by the synthetic model. Both symbols were not detected by the standard model.

**Figure 10:**
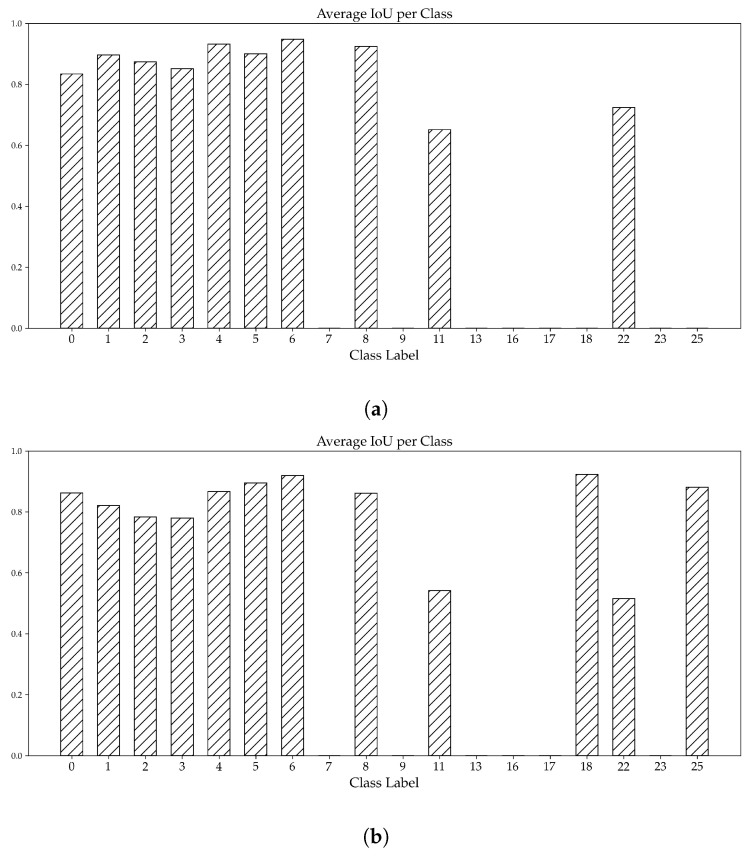
Class-averaged intersection-over-union values, calculated from the detections of the standard model (**a**) and the synthetic model (**b**). An $\bar{IoU}$ equal to 0 correspond to classes with zero detections. Notes: Only classes that occur at least once throughout the inference dataset are presented. To avoid clutter, all versions of the same symbol have been merged into one.

To further assess the performance of both models, the class-averaged intersection-over-union ($\bar{IoU}$) of both models is presented in Figure 10. The performance of both models is generally similar, with $\bar{IoU}$ values ranging from ∼50% to ∼95%. The lowest of those values corresponds to the stairs symbol’s (symbol 22) detections of the synthetic model, highlighting the overfitting issue discussed at the start of this section. For the standard model, the symbol class with the lowest $\bar{IoU}$ is symbol 11. It should be noted that $\bar{IoU}$ values equal to 0 are a result of the definition used in this study, where each undetected symbol in a plan is given $\bar{IoU}$ value of 0.

### 4.5. Inference—CF Model

**Figure 11:**
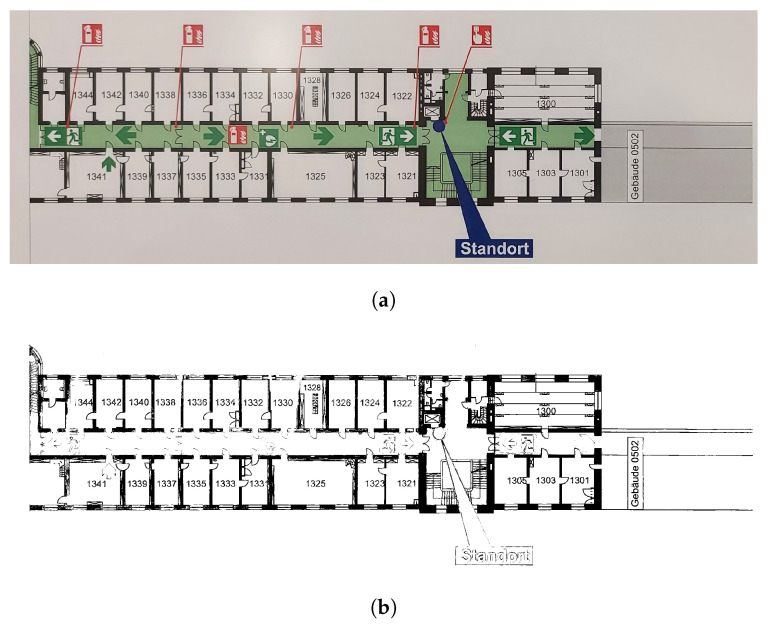
Visualization of the input and output of the CF model, where the input, an emergency floor plan image, is presented in subfigure (**a**), and the output, an image comprising the walls found in the input image, is presented in subfigure (**b**). Both the input and output images are RGB images of the same shape: [inputwidth,inputheight,3].

A sample output of the CF model is presented in Figure 11, where, given an image of an emergency floor plan as input, the model outputs an image comprising the walls found in the input image, i.e., the solid, black lines. It can be seen that the model was not able to extract all walls in the input image, failing to correctly capture the walls in the middle of the top edge of the image.

This inconsistency is likely a resultant of the way the dataset was gathered; since all images in the dataset are photographs of emergency floor plans, the lighting notably varies from one plan to another. Furthermore, the model’s output is extremely sensitive to its hyperparameters, such as the number of clusters used in the preprocessing step, or the binary thresholding value.

Despite these drawbacks, the model is quite simple, comprising a K-clustering algorithm and a non-trainable color filter. While a UNet may be a more appropriate choice for this task, it requires a labeled dataset to train. Since, to the authors’ best knowledge, there are no open-source datasets for emergency floor plans, the CF model presented in this study was therefore deemed a more appropriate choice. Additionally, the utilization of UNets for the purpose of floor plan segmentation is extensively documented in the literature, as mentioned in Section 1.

### 4.6. Clean Plan

**Figure 12:**
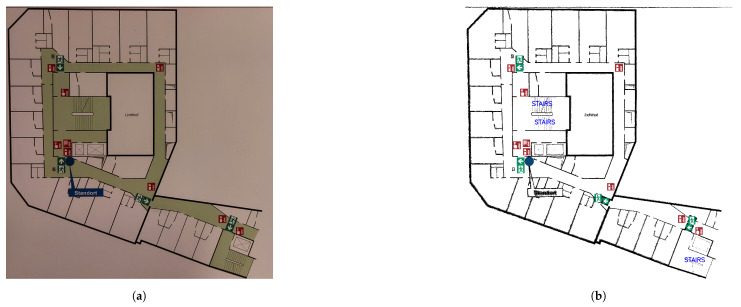
A comparison between an example input to the pipeline presented in this study: a cropped emergency floor plan image (**a**), and the corresponding output of the pipeline: a clean plan, i.e., a emergency floor plan image that includes only the original emergency symbols, staircases and walls (**b**). All symbols were detected using the COD model (the synthetic model, in this case). The walls were extracted using the CF model.

A representative output of the presented pipeline is showcased in Figure 12, displaying a refined emergency floor plan that exclusively incorporates the emergency symbols, staircases and walls from the original plan. This clean plan was generated by combining the outputs of both the COD and the CF models.

### 4.7. Limitations

**Figure 13:**
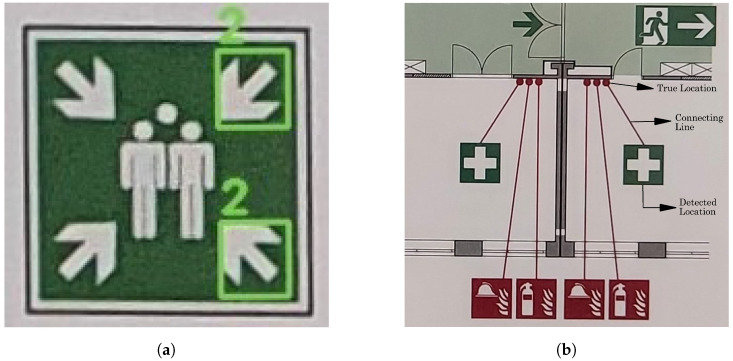
Visualization of the limitation of the presented category-specific object detection (COD) models. (**a**) Incorrect detection of symbols with similar geometries. (**b**) The “true location” problem, where a symbol’s location in the plan is different from its actual location.

A common limitation observed while analyzing the detections of both COD models, is their inability to reliably detect symbols with similar geometries. An example is shown in Figure 13a, where the arrows contained within symbol 6 resemble symbol 2, and are therefore detected as such by both the synthetic and standard models. For such symbols, a larger dataset will not improve the model’s detections, and additional post-processing techniques may be required.

Another issue is the *true location* problem (Figure 13b). In certain plans, particularly crowded plans, the true location of the symbol is indicated by a solid, small circle. The symbol is then placed elsewhere and connected to this circle via a solid line, hereby called the connecting line.

While both COD models are able to detect the true location circles and their corresponding symbols, the connecting line is not detected and, hence, the symbols cannot be mapped back into their original location. The connecting lines generally vary in thickness, shape and color, and hence can be difficult to accurately detect.

Such heterogeneity is also present in the symbol denoting one’s current location, which is marked on every plan. Typically, it is a circular symbol of a consistent color. However, there are no prescribed guidelines for its style; in 403 plans, multiple versions of this symbol were found, differing in shape and color. This inconsistency poses as a challenge in finding a general method to reliably detect it.

Finally, it is worthwhile noting that all the emergency floor plan images used within this study have been manually cropped, removing any additional sections (with the exception of the actual plan) such as the legend. However, these additional sections can include further valuable information. For instance, they may provide details regarding the building’s location in the frequently displayed site plan or convey information about the drawing scale.

## 5. Concluding Remarks

In this study, a framework was created to detect emergency symbols and extract all walls from emergency floor plans. Two models, the COD model—a Faster R-CNN neural network—and the CF model—a color filter with k-means clustering as a pre-filter—were used as part of this framework. The results of both models were than mapped onto one another to create a clean plan: a high-resolution, digitized version of an emergency floor plan, which includes only the emergency symbols and the architectural elements of a plan.

Since, to the authors’ best knowledge, no open-source emergency floor plan datasets exist, a new dataset was collected, consisting of 403 German emergency floor plans. These plans were labeled using a combination of manual labeling and the semi-automatic labeling pipeline described in Section 4.1.2. Furthermore, a larger, synthetic dataset was created using the workflow described in Section 4.2.

Both datasets were used to train two versions of the COD model, namely the standard and synthetic models. These models were tested using 83 emergency floor plan images, and their predictions were compared to a reference benchmark. The two models exhibit comparable performance for common symbols, i.e., symbol classes that occur more than 300 times within the inference dataset. For rarer symbol classes, the synthetic model was more reliable, correctly identifying half the number of two rare symbol classes, symbols 18 and 25. Both symbol classes were not detected by the standard model.

The synthetic model’s ability to detect both symbols suggests that this pipeline can be used on other rare symbols. The steps detailed in Section 4.2 can therefore be used to train for additional symbols, instead of collecting additional emergency floor plans containing such symbols. The relatively lower effort is the main advantage of this idea, since, in addition to the variability in the shape of emergency symbols, no open-source dataset exists for emergency floor plans.

The limitations associated with the presented framework have been identified, namely the incorrect identification of similar symbols, the “true location” issue, the inconsistency in certain symbols’ design, and manual cropping. All limitations were discussed in Section 4.7. Of those limitations, the authors believe that the “true location” problem, i.e., mapping the COD model’s prediction to the symbol’s actual location, is the most crucial. A semantic segmentation model could aid in detecting the “connecting lines”; however, the variability in the lines’ color and thickness could pose as a potential challenge.

## Figures and Tables

**Table 1:** Loss functions used while training the Faster R-CNN model, where RPN is the Regional Proposal Network.

Network	Parameter	Loss Function
RPN	Class	Cross Entropy (binary)
RPN	Bounding Box	Smoothed $L_{1}$
ResNet-50-FPN	Class	Cross Entropy (normal)
ResNet-50-FPN	Bounding Box	Smoothed $L_{1}$

**Table 2:** Lower and upper color limits used to create the color masks in HSV space. All masks were merged together using the binary OR operation.

Mask	Upper Limit	Lower Limit
First Red	[0,50,50]	[20,255,255]
Second Red	[160,50,50]	[180,255,255]
Green	[25,50,50]	[100,255,255]
Blue	[90,50,50]	[170,255,255]

**Table 3:** Transformation operations applied to the training dataset as part of the data augmentation step, along with their corresponding probabilities (in percentage). The transformations are presented in the order they were applied within the code.

Transformation	Probability (*p*)
Horizontal Flip	50
Random Rotation (90°)	50
Motion Blur	20
Median Blur	10
Blur	10

**Table 4:** Specifications of the machine used during inference of both the synthetic model and the standard model.

Component	Details
GPU	Nvidia Quadro RTX 8000
CPU	AMD Ryzen Threadripper 3990X 64-Core Processor
RAM	251.4 GiB

## Data Availability

The datasets compiled within the duration of this study is available upon request from the corresponding authors.

## Appendix A

**Table A1:** List of symbols used in the project.

Label (Ger)	Label (Eng)	Numeration	Symbols
Standort	Location	0	
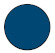
Standort	Location	0_4844-2	
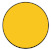
Feuerlöscher	Fire extinguisher	1	
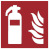
Feuerlöscher	Fire extinguisher	1_4844-2	
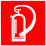
Richtungsangabe	Direction	2	
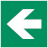
Richtungsangabe	Direction	2_4844-2	
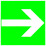
Notausgang	Emergency exit	3	
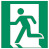
Druckknopfmelder	Push button alarm	4	
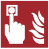
Druckknopfmelder	Push button alarm	4_4844-2	
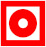
Erste Hilfe	First aid	5	
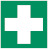
Sammelstelle	Assembly location	6	
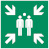
Sammelstelle	Assembly location	6_4844-2	
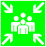
Notruftelefon	Telephone	7	
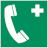
Wandhydrant	Wall hydrant	8_4844-2	
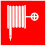
Mittel und Geräte zur Brandbekämpfung	Fire blanket	9	
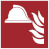
Mittel und Geräte zur Brandbekämpfung	Fire blanket	9_4844-2	
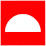
Fahrbarer Feuerlöscher	Mobile fire extinguisher	10	
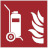
Symbolposition	Symbol location	11	
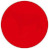
Symbolposition	Symbol location	11_1	
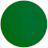
Augenspueleinrichtung	Eyewash device	12	
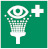
Automatisierter externer Defibrillator (AED)	Defibrillator	13	
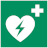
Notdusche	Emergency shower	15	
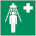
Notausstieg	Escape hatch	16_4844-2	
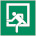
Notausstieg mit Fluchtleiter	Escape hatch with emergency ladder	17	
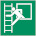
Rettungsausstieg	Rescue exit/mooring	18	
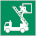
Notausgang für nicht-gehfähige oder gehbeeinträchtigte Personen	Barrier-free emergency exit	19	
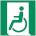
Vorläufige Evakuierungsstelle	Temporary evacuation point for impaired persons	20	
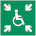
Feuerleiter	Fire ladder	21	
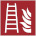
Feuerleiter	Fire ladder	21_4844-2	
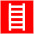
Treppe	Staircase	22	
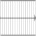
Krankentrage	Stretcher	23	
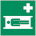
Arzt	Doctor	24	
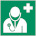
Arzt	Doctor	24_4844-2	
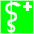
Auslösung RWA	SHEV triggering	25	
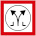
Warnung vor elektrischer Spannung	Dangerous electrical voltage	26	
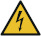
Brandmeldetelefon	Emergency phone	28	
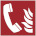
Brandmeldetelefon	Emergency phone	28_4844-2	
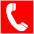
Brandschutztuer	Fire safety door	29	
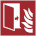
Notausgangsvorrichtung, die nach Zerschlagen einer Scheibe zu erreichen ist	Emergency exit device, which can be reached after breaking a pane	30	
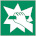
Fest eingebaute Feuerlöschmittel-Batterie	Fixed fire extinguishing battery	31	
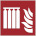
Tragbare Schaumlösch-Einheit	Portable foam extinguishing unit	32	
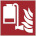
Wassernebelrohr	Water fog pipe	33	
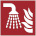
Fest eingebaute Feuerlösch-Einrichtung	Fixed fire extinguishing unit	34	
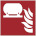
Fest eingebaute Feuerlösch-Flasche	Fixed fire extinguishing bottle	35	
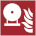
Auslösestation fuer Raumschutz	Sounding station for room protection	36	
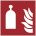
Feuerlöschmonitor	Fire extinguishing monitor	37	
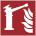
Warnung vor feuergefährlichen Stoffen	Fire hazard warning	39	
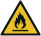
Nothammer	Emergency hammer	40	
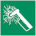
Medizinischer Notfallkoffer	Emergency medical kit	41	
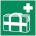
Wiederbelebungsgerät	Resuscitator	42	
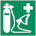
Warnung vor brandfördernden Stoffen	Warning against flammable substances	43	
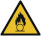
Warnung vor Gasflaschen	Warning against gas cylinders	44	
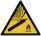
Fluchtretter	Escape rescue device	45	
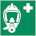
Richtungsangabe	Direction	46_4844-2	
